# Building Community-Based Approaches to Systemic Reform in Mathematical Biology Education

**DOI:** 10.1007/s11538-020-00781-4

**Published:** 2020-08-08

**Authors:** Olcay Akman, Carrie Diaz Eaton, Dan Hrozencik, Kristin P. Jenkins, Katerina V. Thompson

**Affiliations:** 1 Digital and Computational Studies, Bates College, 2 Andrews Rd, Lewiston, ME 04240 USA; 2 Center for Collaborative Studies in Mathematical Biology, Illinois State University, Normal, USA; 3 Mathematics and Computer Science, Chicago State University, Chicago, USA; 4 Bioquest Curriculum Consortium, Germantown, USA; 5 University of Maryland, College Park, USA

**Keywords:** Biology, Mathematics, Pedagogy reform

## Abstract

Starting in the early 2000’s, several reports were released recognizing the convergence of mathematics, biology and computer science, and calling for a rethinking of how undergraduates are prepared for careers in research and the science and technology workforce. This call for change requires careful consideration of the mathematical biology education system to identify key components and leverage points for change. This paper demonstrates the wide range of resources and approaches available to the mathematical biology education community to create systemic change by highlighting the efforts of four community-based education reform organizations. A closer look at these organizations provides an opportunity to examine how to leverage components of the education system including faculty, academic institutions, students, access to resources, and the power of community.

## Introduction

*System: any group of interacting, interrelated or interdependent parts forming a complex and unified whole with a specific purpose* (Kim 1999).

Starting in the early 2000’s, several reports were released that recognized the convergence of mathematics, biology, and computer science (National Research Council 2003, 2013; AAAS 2011; AAMC-HHMI 2009) and called for a rethinking of how undergraduates are prepared for the science and technology workforce. A common theme of these reports was an emphasis on cross-disciplinary collaboration among mathematicians, biologists, and computer scientists in education reform at the undergraduate and graduate levels. Computational biology, quantitative biology, and mathematical biology have emerged as interdisciplinary fields of study, taking their place in academic research and the general curriculum. Many professional organizations and federally funded institutes have arisen to support mathematical biology research and education, including the Society for Mathematical Biology, the National Institute for Mathematical and Biological Synthesis, the Mathematical Biosciences Institute, and the Mathematical and Theoretical Biology Institute. All of these groups have integrated education initiatives into their portfolio of activities and have been influential in promoting cross-disciplinary educational efforts. NSF has funded many grants exploring ways to improve mathematical biology education, and quantitative skills are recognized as critical for all biology students (AAAS 2011).

Despite these efforts, mathematical biology education has not yet achieved its full potential, in part because it represents a complex system in a dynamic and rapidly changing landscape. Historically, biology has been a descriptive science, rather than a quantitative science. Both biology faculty and students may find themselves unprepared for the ubiquitous use of quantitative methods and the rapid development of new quantitative methods (Karpakakunjaram and Jenkins 2019). Math anxiety, rooted in a fixed mindset about mathematical skills, creates an additional barrier to effective teaching and learning of mathematical biology (Boaler 2015). Given the relatively recent emergence of computational, quantitative, and mathematical biology, the incredible growth in biology and computational research, and pedagogical challenges, it is not surprising that the systemic organization supporting mathematical biology education is itself evolving quite quickly.

**Fig. 1:**
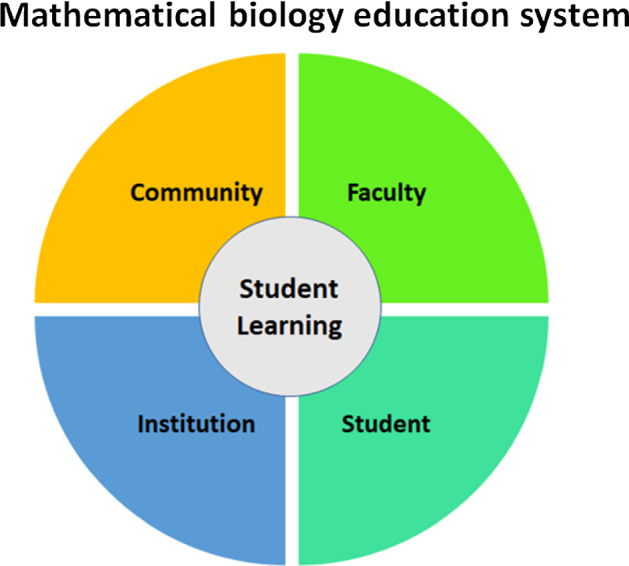
Components of a broader postsecondary education system which work together to enhance student learning in mathematical biology

Education focused grassroots efforts arising in the biomathematical and quantitative biology communities have contributed to filling these gaps in the mathematical biology education system (see Fig. 1). These projects have encompassed expertise in mathematics and biology education reform, development of education materials, and professional development. Each organization has taken a different approach, designed to address somewhat different, but overlapping, aspects of the system. Four such grassroots-founded organizations are described in this paper: BioQUEST, MathBench, QUBES, and IBA. The four education reform organizations highlighted have taken different approaches to addressing systemic change, with the common goal of transforming mathematical and quantitative biology to enhance student success. The BioQUEST Curriculum Consortium is a transformative, collaborative community empowering educators to drive innovation in STEM education. MathBench is an online collection of educational modules that aim to reinforce biological concepts, increase math literacy, and prepare students for more complicated mathematical approaches in upper-level courses. QUBES is a community of mathematics and biology educators who share their resources and methods for preparing the next generation biomathematics scholars to successfully use quantitative approaches to solve real-life complex biological problems. Finally, the Intercollegiate Biomathematics Alliance (IBA) is a diverse multi-institutional consortium that sponsors synergistic activities of biomathematics researchers, educators, and students in order to promote educational and research opportunities. A unifying theme of these grassroot efforts is to form and sustain a productive and efficient environment where a diverse community of faculty and students are supported and provided with tools to achieve their respective goals. These organizations intersect with the mathematical biology education system at several key points including faculty, students, institutions, and educational resources. An overview of how these four reform organizations have contributed to mathematical biology education reform may provide ideas and encouragement for others who seek ways to improve mathematical biology education. The organization of this manuscript is historical—from the earliest founded initiative to the most recent. For each organization, the spark or inspiration is described, as well as the organizational philosophy, and how the community benefits and participates. An important aspect of these efforts is a focus on community building that serves to increase access to resources for both faculty and students, and broaden the inclusion of a wider range of institutions and individuals. In the conclusion, we reflect on the impact these projects have had on improving the system of undergraduate biomathematics education, where challenges remain, and where the community can contribute to future reform efforts.

## Organizations

### BIOQUEST

BioQUEST arose from a meeting of the minds between John Jungck and Nils Peterson on a walk through Torrey Pines, California, in 1986. Their idea that students should practice and experience science as scientists do was crystalized in the 3P’s Philosophy of “Problem Posing, Problem Solving, and Peer Persuasion” (Peterson and Jungck 1988). This philosophy is deeply rooted in a recognition of the holistic experience of learning, addressing the interdisciplinary nature of biology, and the critical role of inclusion and social relevance in student success. Funding from the Annenberg/CPB Project, Apple Computers, Inc., the Foundation for Microbiology, and the Center for Biology Education at the University of Wisconsin-Madison supported the initial exploration of this educational approach. That educational experiment has lasted over thirty years and is still going strong.

The BioQUEST Curriculum Consortium brought together an interdisciplinary community of educators who developed software simulations to engage students in actively exploring biological phenomena through the new technology of personal computers. These initial educational modules included the Genetics Construction Kit, which despite its age is considered by many to be among the best simulations for teaching genetic concepts. The modules were made available on CDs, another cutting edge technology, in the BioQUEST Library. In 1996, BioQUEST materials moved to a new platform—the world wide web. Development of new educational materials was supported by the BioQUEST Summer Workshops, which at the time were nine very long days of intense, collaborative work. Participants often referred to the experience as “biology boot camp,” but they kept coming back. Participants continued to develop new educational materials, leveraging the use of easily accessible tools such as Excel to engage students in quantitative biology, especially in bioinformatics and evolutionary biology. In the late 1990s, BioQUEST expanded beyond simulations to include professional development around pedagogies that supported the 3P’s Philosophy such as the Investigative Case-Based Learning project, a variation of Problem-Based Learning. Recent research in effective educational pedagogies has demonstrated the efficacy of many educational practices BioQUEST has promoted over the years (National Research Council 2015), and BioQUEST continues to serve a translational role for the community of practitioners, by demonstrating how to bring research-based practices into the classroom. Another way that BioQUEST has led the way in educational reform is the commitment to generating open education resources. Most of the materials generated by the BioQUEST community were made available as open education resources, which led to broad use and adaptations that have kept the materials relevant.

The impact of BioQUEST on biology education has been recognized in various ways. In 2010, BioQUEST received the American Society for Cell Biology (ASCB) Bruce Alberts award for Excellence in Science Education. In 2014, NSF funded a study of persistent, impactful professional development organizations in STEM education reform (Kezar and Gerhke 2015). This research study identified BioQUEST (as well as SENCER, POGIL and Project Kaleidoscope) as “communities of transformation,” in that they encouraged community members not only to improve their practice but to challenge the accepted norms in a search for better solutions. The transformative impact of BioQUEST is evident in the enthusiastic community members who collaborate on papers, grants, and projects and support one another in developing educational materials and practices that challenge both faculty and students to go beyond traditional approaches. BioQUEST is a highly collaborative organization in which multiple funders, professional societies, and creative individuals have come together to address educational challenges. Funders over the years have included NSF, NIH, HHMI, and Annenberg. BioQUEST has collaborated with the National Evolutionary Synthesis Center (NESCent), the National Institute for Mathematical and BIological Sciences (NIMBioS), mathematics and biology professional societies, projects such as Shodor, and offered workshops at dozens of universities and colleges around the world.

A typical BioQUEST project is ESTEEM, which leveraged Excel spreadsheets to introduce students to quantitative models of biological phenomena. ESTEEM was a collaborative, interdisciplinary project that emerged from prior work in bioinformatics (BEDROCK) and partnerships with Shodor, MAA, and HHMI. Participants in BioQUEST workshops were introduced to ESTEEM modules, and invited to generate their own. All ESTEEM modules are publicly available, and many are still used in the classroom. This commitment to open access and engagement of community members to both develop, adapt, and use materials is a hallmark of BioQUEST’s approach. Another example is BioQUEST’s Investigative Case-Based Learning which focused on the pedagogical approach faculty use to engage students in thoughtful analysis of biological problems. This project led to a series of workshops that reached faculty across the US and around the world. The Science Cast Net, an NSF funded RCN UBE, is the current iteration of this project. Science Case Net brought together several organizations promoting case based pedagogies and supports several focused case development groups including the High-throughput Discovery Science& Inquiry-based Case Studies for Today’s Students (HITS), and the Molecular Case Net.

Today, BioQUEST continues to explore frontiers in science education and promote practices that engage all students in a relevant and meaningful learning experience. BioQUEST has become a 501(c)3 non-profit organization to continue productive collaborations addressing both emerging and persistent educational challenges, through professional development, new educational materials, pedagogical approaches, and policy leadership. Faculty may join the BioQUEST community at the annual Summer Workshop, and through collaborative projects such as QUBES and the Science Case Network. Together, this community continues to delve into emerging influential disciplines such as data science and tackle enduring challenges in providing all students with equitable, inclusive learning opportunities.

### MathBench Biology Modules

MathBench Biology Modules (mathbench.umd.edu) were created at the University of Maryland (UMD) in 2004, against a backdrop of increasing emphasis on quantitative and interdisciplinary approaches to biology. The National Research Council’s (2003) Bio2010 report National Research Council (2003) had just been published, and it resonated with the growing desire of UMD biological sciences faculty members to introduce more quantitation in upper level courses to prepare students to contribute to the biological sciences research enterprise. These faculty members, led by Bill Fagan and Avis Cohen, had built their research programs at the interface of biology and mathematics, and they recognized that undergraduate students in the biological sciences needed greater exposure to the use of math in biology at every level of the curriculum. This view was reinforced by institutional data showing that success in introductory biology courses was closely linked to proficiency in mathematics.

MathBench was conceived as a series of self-contained, online modules that complemented traditional lecture- and laboratory-based introductory biology courses. From its inception, MathBench incorporated three strategies rooted in the science education literature (Nelson et al. 2009):

- Use of an informal tone, humor, story lines, and connections to real life to dispel math anxiety
- Use of interactive elements to promote student engagement and active learning
- Incorporation of individualized feedback to students to provide them with some control over the amount of instruction and practice they receive while completing the modules

Module topics (e.g., logarithms, basic statistics, and graphing) were identified via meetings between the project team (primarily lead developer Kären Nelson) and faculty members who taught the first four courses required for the biological sciences major. All faculty recognized the need for students to appreciate the relationship between mathematics and biology and develop basic proficiency in applying mathematics to solve biological problems, but not all of them were comfortable teaching quantitative concepts. Therefore, the modules were designed to be done as homework, outside of class time. In some cases, the content of the modules was revisited by the course instructor during lecture or laboratory meetings, but in other cases, it was purely supplementary material.

Initial financial support for the creation of MathBench modules came from a Howard Hughes Medical Institute Undergraduate Science Education Program grant to the University of Maryland, and the project received support for subsequent expansion and dissemination from two National Science Foundation Course, Curriculum, and Laboratory Improvement grants. This additional funding allowed MathBench to launch a collaboration with James Sniezek, then a biology faculty member at Montgomery College, a nearby 2-year institution, which in turn sparked the development of several new modules geared to the community college audience.

While MathBench was first developed specifically for UMD (and later Montgomery College) students, because the modules were freely available on the web, they quickly gained an international audience. Recently, the project has partnered with a consortium of seven Australian universities, which received funding from the Australian Government’s Office for Learning and Teaching to adapt 27 of the modules for use in Australian universities www.mathbench.org.au. This project also brought an infusion of mathematical expertise through the participation of mathematics faculty members from the Australian partner institutions.

In the early years of the project, there was little effort to establish a community of MathBench users. Each module had been created for a particular course based on the recommendations of the instructor, and each instructor had a particular vision for how the module would be integrated into their course. A social networks analysis of several UMD curriculum projects that aimed to strengthen the quantitative skills of biological sciences students showed that, rather than being a tightly interconnected network, the MathBench project resembled spokes of a wheel, with each participating faculty member connected only to the module developer (Thompson et al. 2013).

That isolation has diminished with the increasing prominence of other organizations that share a commitment to quantitative biology education. Collaboration with these organizations, particularly the BioQUEST Consortium and QUBES, has been key to the wider dissemination of MathBench and our recent efforts to establish a community of MathBench users. The BioQUEST Consortium, through its annual summer workshops, has provided a venue for recruiting and training new MathBench users. QUBES has been instrumental in providing the necessary networking infrastructure to facilitate long-term interactions among MathBench users. In Fall 2018, MathBench sponsored a QUBES Faculty Mentoring Network to provide professional development for faculty members seeking to implement the modules in their courses. This resulted in the creation of instructional materials that are being shared with the MathBench user community through the QUBESHub cyberinfrastructure. In future years, we hope to preserve the existing modules for continued use, as well as develop new modules that target additional concepts and skills identified by our community of users.

The evolution of MathBench over the years is very much reflective of changes in the national landscape of STEM education. In its early years, the project was somewhat siloed, both with respect to individual courses and campuses. As the community of adopters has broadened, we have gained greater insight into how best to implement the modules and promote a deeper understanding of quantitative biology. It is now clear, for example, that students gain little from the modules if they are perceived as an add-on rather than an integral component of the course. The highest learning gains are seen when the modules are tightly woven into the fabric of the course and the skills reinforced by the modules are required for success in the course (Karsai et al. 2015). It is also clear that student attitudes towards the utility of mathematical approaches to biology are slow to change, and therefore need to be reinforced at multiple points in the curriculum (Thompson et al. 2010).

### QUBES: Quantitative Undergraduate Biology Education and Synthesis

Spurred by the interest in mathematical biology research and subsequent national STEM Education reports and National Science Foundation investment in mathematical biology education program building, many educational institutions simultaneously began to innovate in the education interface between mathematics and biology. While the flurry of attention to mathematical biology was welcome, there were re-occurring coordination issues between these siloed groups and individuals. Information about curricula was shared primarily at select sessions across multiple subdisciplinary professional societies. Faculty created materials and posted to their own websites, where it might never be used or known by faculty at other institutions. Active, but underfunded, groups of professional societies devoted to mathematical and quantitative biology education were not equipped to coordinate national scale projects to coordinate and disseminate curricula. QUBES (Quantitative Undergraduate Biology Education and Synthesis) was founded in 2013 in response to the recognition that many of these individuals and groups were establishing an emerging field of quantitative biology education, but separately and without overall coordination or guidance on best practices.

The grand idea behind QUBES was to develop a cyberinfrastructure (Hub) to provide coordination for these multiple education initiatives, but with the key feature that we recognized all professional societies and individuals had to work together to achieve this common goal (Consortium). QUBES as an idea was initially funded as an exploratory NSF Research Coordination Network for Undergraduate Biology Education. The vision of the leadership was that

*“The QUBES hub will bring together a community of quantitative biology educators that can create, share, and assess curricular resources using a dynamic online network that reaches across departments, colleges, institutions, and countries.”*

The proposal laid the framework for a social-cyberinfrastructure to move forward collective action on undergraduate biology education.

QUBES invited several stakeholders and potential collaborators representing biostatistics education, mathematical biology education, quantitative biology education, professional societies (such as Ecological Society of America, BioSIGMAA, Society for Mathematical Biology, the American Association for the Advancement of Science, and Association of Biological Laboratory Educators), and professional development (such as BioQUEST) to a meeting at NIMBioS. At this meeting, the network carved out a central and shared goal around quantitative biology education and agreed upon priority activities. Shortly after, QUBES was invited to participate in a collaborative effort by a team out of Radford, Pittsburgh, and BioQUEST to write an Ideas Lab proposal, which ultimately funded the current QUBES project This evolution of QUBES added a strong component of professional development and also solidified a firmer connection to a diversity of biology professional development societies [for more details see QUBESHub.org (2020)].

QUBES consists of overlapping, but complementary, activity areas:

- Consortium—facilitates collaboration and communication between a broad array of partner projects, institutions, professional societies, and the quantitative biology education community;
- Hub—the cyberinfrastructure needed to support QUBES activities from collaboration spaces and websites to cloud-run computing and modeling tools;
- Open Educational Resources—the Hub hosts and links to curriculum and software from both partner projects and the community sourced that can be freely used, modified, and shared;
- Professional Development—Faculty Mentoring Networks provide faculty support from peers and experts throughout the semester while they implement new educational resources and practices; and
- BioQUEST—grounds our virtual community with a jointly hosted QUBES-BioQUEST summer quantitative biology education workshop each year in addition to other support activities across the areas.

In addition to the five directors of these activity areas, QUBES grant funds support a web developer, a web designer, a postdoctoral research student, as well as project managers and contractors (See “About us” at QUBESHub.org). Together, we have a shared vision to support educators and advance an inclusive quantitative biology through the power of collective impact.

**Fig. 2:**
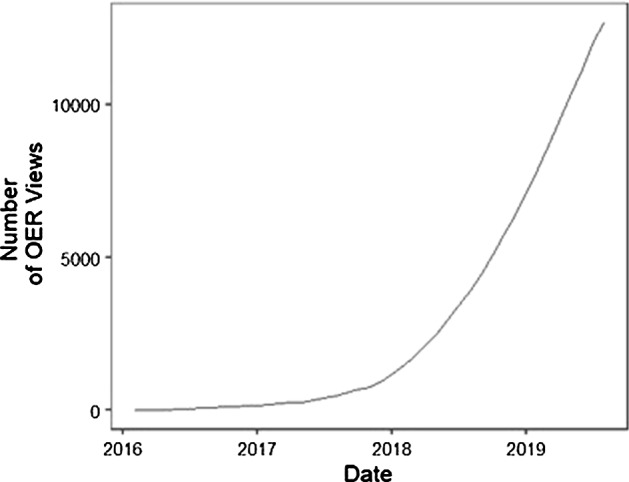
Number of views of OERs on QUBES Hub (Figure by Dr. Elizabeth Hammond). QUBESHub was soft-launched in 2015 and publicly launched in 2016. QUBES also hosted the first Faculty Mentoring Networks in 2016 and the first joint QUBES-BioQUEST meeting in 2016

As of June 1, 2020, the QUBES Consortium has worked with over 100 partners and collaborators over the six years the RCN and Ideas Lab projects have been funded. QUBESHub now hosts or connects to 1134 resources (See Fig. 2), 46 open-source cloud-run tools, hosts 436 partner websites and educational project management pages, and has welcomed 11,928 registered users. Since the website launch, QUBES has partnered to help deliver semester-long virtual professional development to over 700 Faculty Mentoring Networks participants who are adopting quantitative resources, tools, and Open Educational Resources (OERs) for their classrooms—nearly half of those in the last two years. QUBES also serves approximately 80–100 people each year through our summer workshop collaboration with BioQUEST and collaborates on a number of conferences, including an NSF INCLUDES funded conference on environmental data science with NEON which reached nearly 200 on-site and virtual participants (Lauer et al. 2020). QUBES leadership and QUBES Ambassadors also routinely give talks and workshops at various professional meetings, reaching an even broader audience.

The community QUBES serves is diverse, representing a variety of institution types and around the world (though, primarily from the United States) and a variety of disciplines which act at the interface of biology and the mathematical and computational sciences. In terms of impact on individuals, we are now also beginning to see cumulative effects, for example, Faculty Mentoring Network participants report this professional development activity in their tenure and promotion portfolios, and colleges’ public relations departments highlight mentors who have led Faculty Mentoring Networks. In terms of project impacts, stories like MathBench above are one of many use cases in which projects have been able to expand their outreach into new communities and facilitate new adaptations of their resources to meet emerging or institutional needs. QUBES now routinely contracts its services, such as conference support, web design, publication systems, and Faculty Mentoring Networks to professional societies and projects looking for similar success stories.

QUBES has also invested in research and evaluation of services and products, in order to optimize practices for the shifting technological, and educational and future workforce landscapes. As a result, QUBES has been on the leading edge of providing professional development in data science education for biology. QUBES is also a leader in social-cyberinfrastructure innovation, with an open educational resource system that harnesses the power of virtual communities. Community building and Consortium efforts lead in part by QUBES, such as EDSIN and SCORE, encourage discussion specifically about supporting historically underserved communities. We have also been working with colleges and universities with Howard Hughes Medical Institute Inclusive Excellence grants. While these efforts smartly tap into a growing “new majority,” it is also crucial for our QUBES community to embrace a broader community in order to do our best work and to amplify the incredible work done within and by these communities. As a leader in education on an emerging front, QUBES takes seriously the responsibility to engage with and not just for underserved colleagues and to do our part to help close disparity gaps in STEM education. These actions are aligned with a commitment to shape a quantitative biology education that is open, ethical, inclusive, and universal.

QUBES endeavors to provide a place for all educators to find both resources and community. It is meant to be community driven, and to support this community to do its best work. While initially NSF funded, QUBES requires the continued support of community members like our readers to keep QUBES free and up to date with emerging community needs and web technologies. Being part of the QUBES community functionally means creating an account, downloading a resource, joining a group or Faculty Mentoring Network, contributing resources, and/or simply signing-up for the newsletter. For organizations, partnering with QUBES can mean sharing information across networks, partnering to offer a Faculty Mentoring Network, and/or contracting services.

The offering of contract services hints to issues on sustainability as QUBES grapples with its commitment to offer professional development and resources for free to the instructional community as our NSF funding ends. QUBES contracts go in part to support web hosting services, but also to support the human infrastructure often invisible behind online community platforms. These virtual services have become even more important in light of online shifts due to COVID-19. We are fortunate to also have attracted supplemental funding from the Hewlett Foundation for the upcoming year. In five years, we hope that QUBES is still providing an open, accessible, active, online community platform that reaches all people with tools, resources, and support relevant to the new challenges in the biomathematics education and research community.

### Intercollegiate Biomathematics Alliance (IBA)

For many years, higher education has been suffering under the adverse effects of diminishing fiscal resources due to the changing political and social attitudes towards education. Overall, states have been consistently decreasing the funding for public state higher education institutions (Mitchell et al. 2018). As a result, higher education institutions have had to balance budgets by reducing faculty, limiting course offerings, and in some cases, by closing campuses. Furthermore, surviving academic programs have found themselves operating under limited resources, which made access for researchers and educators increasingly difficult. Mathematical biology is not an exception to these trends. Especially, since this is an emerging interdisciplinary field, practitioners are more vulnerable to intellectual isolation than other fields in mathematics. Naturally, lack of an already-established advanced student intellectual workforce makes scientific progress in the field even harder. Hence, lack of academic resources, lack of faculty expertise, and lack of students, advanced or otherwise, lead us to the consequence of limited curricular innovation in mathematical biology. As expected, the creation of an organization to address these issues is what motivated the formation of the Intercollegiate Biomathematics Alliance (IBA).

**Fig. 3:**
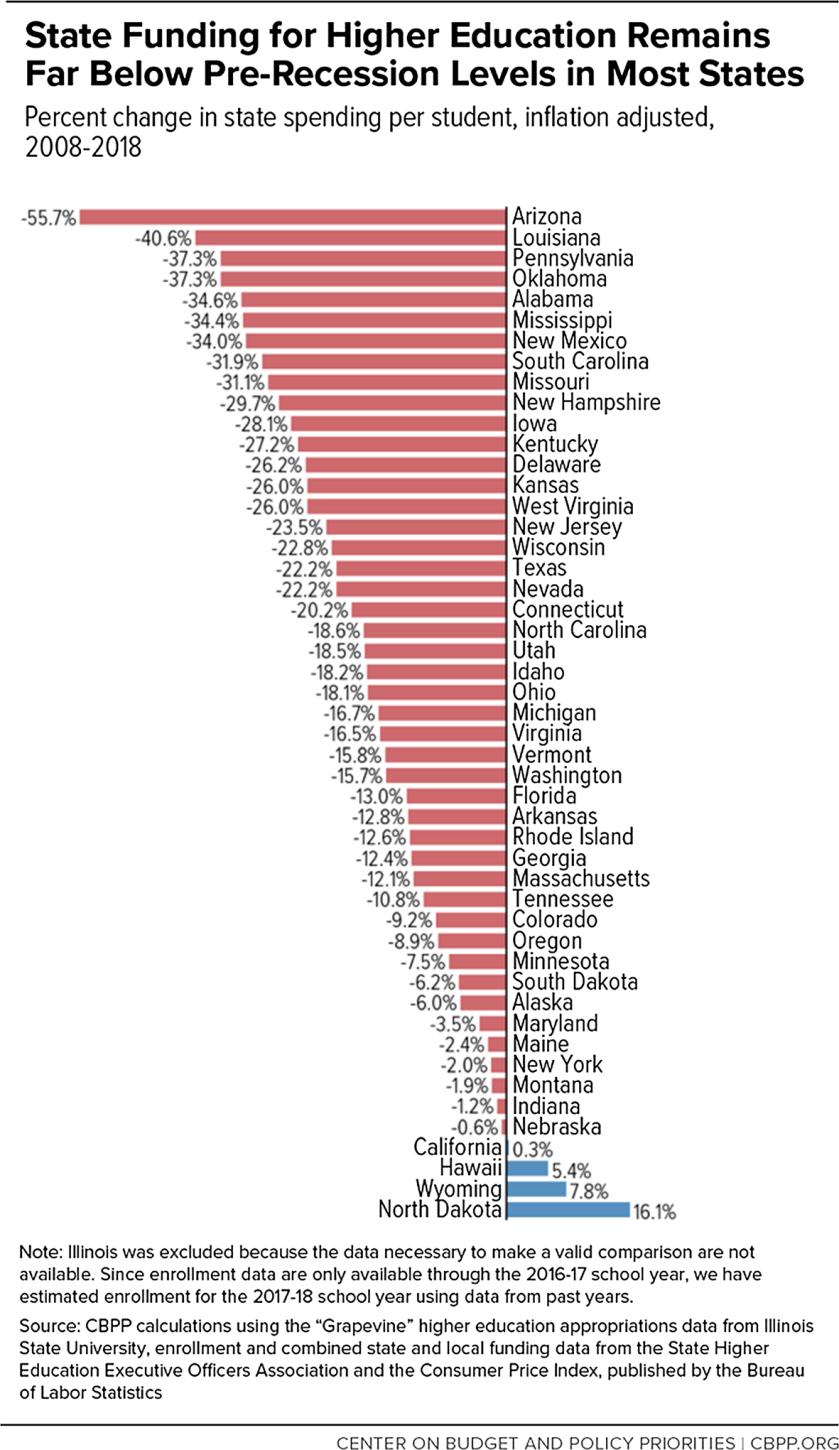
Percent change in state spending per student, inflation adjusted, 2008–2018. Reproduced with permission from the Center on Budget and Policy Priorities (Mitchell et al. 2018)

The initiation of IBA, a comprehensive academic collaborative entity addressing the discipline-specific needs of mathematical biology programs, was founded in early 2014 at Illinois State University (ISU). Originally conceived as a way to facilitate inter-institutional research among the three founding members, the goal of the IBA became to create a small network of universities, all contributing limited funds to a pool from which the research activities of similar collaborating groups may be supported. The early success of this initiative then led the founding members to expand this collaborative structure to other institutions with faculty of similar research interests and needs. As a result, a consortium of dues paying member institutions was formed. In this consortium, each member institution is a stakeholder where their students and faculty have unique access to a wide range of community-based support, as outlined below. In three short years, the IBA has grown from a small network of three universities to a consortium of twelve institutions with diverse size, mission, and demographic make up. As the network grew, the mission and the interests of the members naturally expanded to other scholarly activities such as but not limited to supporting undergraduate research, community curriculum, and an enhanced academic infrastructure. Based on the current goals of the consortium, the IBA states its mission as follows:

*[IBA’s] mission is to provide a platform for expanding access to a diverse network of scholars and resources at the interface of biology, mathematics, and computational science to enhance research and education opportunities for researchers, educators and students.* (IBA 2019)

The following highlight some of the main components of IBA’s mission.

*Cross-Institutional Undergraduate Research Experience (CURE)* Cross-Institutional Undergraduate Research Experience (CURE) is a workshop where IBA member students and faculty research mentors gather during an all-expenses-paid extended weekend to experience the entire process of conducting research in mathematical biology. Faculty Mentors present cutting-edge research projects that they are interested in directing and collaborating on with another faculty. Then, students visit with each Faculty Mentor to decide which project they want to work on throughout the year. The workshop continues with programming and scientific/technical writing boot camps. The workshop also includes presentations on various career directions. Current and former graduate students give presentations on graduate school experience and life as a professional working in a data-analysis related industry. Each day ends with a fun activity; a student versus faculty soccer match and a student versus faculty bowling competition. The CURE workshop without a doubt has proven to be one of the most successful initiatives of IBA. Student and Faculty Mentor involvement and the undergraduate research output, in various formats, have been increasing steadily and substantially in both quality and quantity.

*Biomathematics Education with Applications and Methods (BEAM)* Project BEAM will make funding available to cultivate undergraduate research at member institutions. There are two funding tracks: one that requires institutional matching and one that supports faculty with no access to institutional research funding.

*Partners in Extending Education and Research (PEER)* PEER is an interdisciplinary scholarly support service program that the IBA offers to educators, researchers, and scholars of cross-disciplinary fields. The PEER Program matches a cross-disciplinary researcher with a data scientist, statistician, applied mathematician, or computer scientist with a higher-level data-science expertise than what may be common in other fields. The PEER program has been so successful that the IBA’s host institution now funds a research graduate assistant position exclusively for PEER.

*CLOUD for Layering, Organizing, and Utilizing DATA (IBA-CLOUD)* The IBA houses a high-performance computer. Each node sports a 32 core/64 thread AMD $\hbox {EPYC}^{\mathrm{TM}}$ 7551P processor and RAID array with full-disk encryption for maximum performance and reliability. IBA offers access to members for their research-level computing needs. IBA-CLOUD is already in use at full capacity facilitating cutting-edge research computing for members.

*Graduate Certificate Program* The IBA offers a flexible graduate curriculum in mathematical biology, with offerings through different options, both online and traditional coursework, students have a variety of options for obtaining a certificate in this program. This program’s innovative approach is highly praised by stakeholders.

*Sponsored Journals* The IBA sponsors two high quality open access refereed research journals as a part of its mission of providing avenues to the biomathematics community at large to disseminate their work. The first journal, Letters in Biomathematics, publishes peer-reviewed research articles in mathematical biology and its education as well as review articles in the field. The second journal, Spora, is the only student research oriented journal in the world dedicated to biomathematics. These two journals have filled a niche in the field each in its own unique way. The Letters in Biomathematics was the first biomathematics journal that published biomathematics education research side by side with review and research articles. Spora’s success as a platform for hosting undergraduate research has led to expanding its mission by publishing research conducted by graduate students.

*Other Resources* In addition to the above resources, IBA also offers unique resources to member institutions, such as (1) community curriculum, where students from member institutions may take pre-screened and approved courses offered by other member institutions, (2) faculty research retreats, where faculty members work on collaborative projects, (3) research dissemination support, where members receive financial support for open access, among others. The IBA is a unique concept which is widely regarded as an efficient and productive avenue to foster research and education in mathematical biology. What makes IBA highly efficient and productive is its approach of targeting stakeholders via the institutions in which they belong. Although other initiatives of similar kind with varying missions depend on a volunteer academic workforce, an important building block of IBA without a doubt is its dedicated staff. IBA’s leadership team consists of paid staff members who perform activities covering different parts of its functionality. In addition, IBA’s mission of supporting research and education extends to individual faculty members from non-member institutions. This way, interested and eager researchers and educators also enjoy most of the resources that are afforded to IBA’s institutional members. In fact, this model has proved to be so productive that not only other fields of mathematics but also other disciplines may benefit from a similar approach to research and education. Advancing biomathematics as well as any other scientific field is a multifaceted endeavor whose components are conducting cutting-edge research, educating future scientists, providing platforms to advance the field, providing avenues to disseminate knowledge, and sharing contemporary research tools and expertise. In this context, IBA’s very mission aspires to unify all these components resulting in the highly productive and successful enterprise serving the biomathematical community. Without the IBA’s community-based education and collaborative research philosophy, current member institutions would not have access to the resources that allow them to engage in an active research and education agenda for a rapidly emerging and changing field. Although this community-based education delivery model has started impacting a larger number of programs thanks to the increasing number of institutions joining the consortium, its impact is not yet broad enough to be considered mainstream. The mathematical biology community has an important role in embracing this novel approach in replacing the traditional thinking of “in order to offer a new program, an institution needs to have all the necessary components of this program in place before it can be offered.” Federal or state funding agencies have an important role in supporting this new research and education model as well. Seed funding will allow new institutions to experience the benefits of being a part of this community before committing institutional financial and academic resources.

An important change to the current IBA model which will increase its national profile is the addition of a scientific publishing initiative, called IBA Press. Starting in 2020, the IBA Press will serve the mathematical biology community by publishing and marketing educational and classroom materials created by its stakeholders that are proven to be successful in a single institution but not known in others. Moreover, IBA will acquire and sponsor the international research journal Letters in Biomathematics in an effort to make this well-respected and highly reputable research and education dissemination platform readily accessible to the mathematical biology community.

**Fig. 4:**
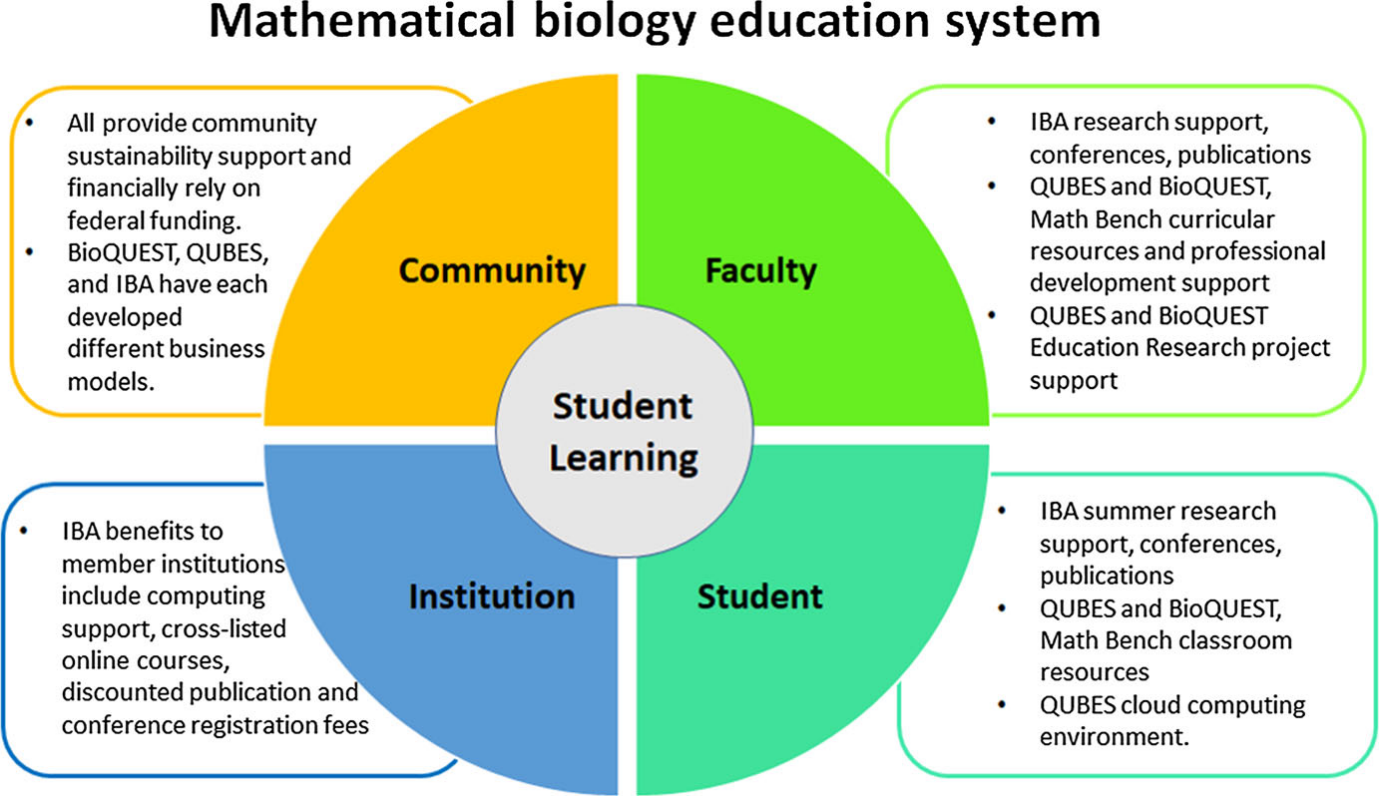
Working together towards community-based interventions on the entire mathematical biology system

## Discussion

*Systemic education reform efforts at the faculty level* A key leverage point for change in this system is faculty. Efforts to influence this system must support both mathematics and biology faculty by filling in gaps in educational resources, promoting effective pedagogical practices, and providing creative, encouraging communities where faculty may stay up to date on their fields (see Fig. 4). The structure of each of the four education reform groups highlighted here reflects the needs of the community and available resources at the time each organization originated. All have focused on supporting faculty efforts and all have a strong community component. Each organization is a grassroots effort, relying on the active engagement of volunteers. All are community focused, and the direction of these projects is driven by the needs of faculty. BioQUEST serves individuals and supports emerging educational projects, and as a result feels similar to a professional society. MathBench focuses on developing curriculum resources with and for instructors and institutional partners, amplifying dissemination through community. The QUBES community consists of individuals, serving them through projects and programs from professional societies, centers, and curriculum projects. IBA offers institutions the power of a collective to leverage access to valuable educational resources. Each organization also supports the growth of their community through professional development, access to resources, and communication about ideas and opportunities. Many educational reform projects begin with the notion that creating materials and making them widely available will be sufficient for widespread adoption of new educational approaches. That strategy, which relies solely on increasing instructor awareness, has proved ineffective (Foote 2014; Henderson and Dancy 2009; Henderson et al. 2011). Providing educational materials is an important component of BioQUEST, MathBench, QUBES, and IBA, but these organizations have evolved to recognize that a more holistic approach is necessary to promote deep and permanent change in educational practices. To achieve these ends, these organizations are highly collaborative and readily form partnerships with relevant educational initiatives and institutions. As an example of this partnering, MathBench has shared resources at BioQUEST’s professional development workshops for many years and has offered a Faculty Mentoring Network through QUBES, and QUBES and BioQUEST are deeply intertwined in efforts to serve the community. These collaborations allow resources to be shared with a broader audience than any single organization could reach, creating a win-win situation for all participating organizations.

On an individual classroom basis, faculty report changing their practices and use of better resources based on exposure to these education projects (Gehrke and Kezar 2016). However, despite the wealth of available resources, faculty struggle to identify and incorporate quality materials on their own. Access to readily available, high quality educational materials can make a substantial contribution to systemic educational reform. The recent interest in OERs is a case in point. Many community colleges and a growing number of four year institutions have moved to adopt OERs to reduce costs for students. However, faculty face several barriers including locating and evaluating OERs (Davis et al. 2009). BioQUEST, MathBench, QUBES, and IBA have addressed the need for educational materials, generating and disseminating materials, promoting adoption across their communities, and supporting the development of new materials by community members. For example, the Quantitative Biology at Community Colleges (QB@CC) a BioQUEST project, utilizes the QUBES infrastructure and services and taps into MathBench resources to provide faculty with OERs as well as professional development supporting their adoption. Promoting and rewarding the generation and use of OERs and other quality educational resources through professional development, sharing information in articles, and recognizing educational materials as publications is an important component of systemic change.

*Systemic education reform efforts at the institutional level* BioQUEST, MathBench, and QUBES have contributed to improvements in individual faculty practices, but institutional change remains a major challenge in systemic reform efforts. Unless change occurs at the institutional level, student experiences will remain inconsistent, and reform efforts will be limited to the individuals or small groups who embrace them. When these faculty leave the institution, the reforms also leave. IBA has a model in which institutions can buy into the association, so there is a direct impact on institutions in addition to its student, individual, and community-level initiatives. The focus of these interventions has been on access to resources and support for research and course delivery. However, it is a reflection of the difficulty of the problem that decades of effort by disciplinary projects has failed to result in substantive institutional change. Despite the impact of BioQUEST, MathBench, QUBES, and IBA on individual practices, there is little evidence of successfully influencing systemic change even at the departmental level (Gehrke and Kezar 2016). However, it is not lost on us that quite often institutional change starts with individual faculty efforts. Eventually these efforts attract others and become the critical mass that is capable of planting the seeds for an institutional change. The combined efforts described on this work have this very potential. Faculty themselves may be caught up in the mixed messages they have received throughout their academic careers, about the value of teaching relative to research pursuits (Brownell and Tanner 2012; Austin 2011). The emphasis on research rather than teaching is deeply embedded in scientific culture, reflected in professional societies and institutional structures. A key leverage point for systemic change is remodeling the academic reward system to recognize the relevance of time spent on teaching, professional development, curriculum development, and course improvements. This kind of transformational thinking is supported by education reform communities such as the four highlighted in this paper. Until teaching, and the continuous improvement of teaching, is respected and rewarded commensurately with research endeavors, faculty have little incentive to commit more time and energy to changing their teaching. A number of national projects are grappling with amplifying the impact of effective reform efforts from the individual level to the departmental level and beyond. Projects, such as Accelerating Systemic Change in STEM Higher Education, are applying the scholarship of change to higher education systems, while others such as NSITE and Bridging to STEM Excellence are bringing together disciplinary educational reform projects to leverage systemic influences through combined resources, knowledge and impacts for institutional change. Professional societies and faculty can support systemic change by advocating for updates to the faculty reward system, which does not recognize the work and risks involved in educational reform (Fairweather 2008).

*Systemic education reform efforts at the student level* The common goal of all education reform efforts, including the four highlighted here, is improved student outcomes. However, this part of the education system is also changing rapidly. The “traditional” student, who moves from high school through a two year or four year degree, has become elusive (National Academies of Sciences, Engineering, and Medicine 2016). Students are moving between institutions in the course of their studies and sitting out semesters or even years. As part of the demographic shift, students are older, have jobs, and family obligations. More students are first generation or veterans. Increasing numbers of traditionally underrepresented students (women, students of color, students with disabilities) are seeking higher education.

These non-traditional students often leave STEM disciplines because the teaching methods are not inclusive, or effective (Seymour and Hunter 2019). The body of knowledge about learning, learning environments, and teaching has grown immensely in the past twenty years (National National Academies of Sciences, Engineering, and Medicine 2000; National Academies of Sciences, Engineering, and Medicine 2018a). This research has led to the development of effective educational practices (National Academies of Sciences, Engineering, and Medicine 2015), and to specific suggestions within biology (AAAS 2011) and mathematics education (National Research Council 2003, 2013). BioQUEST, MathBench, QUBES, and IBA have incorporated this information in their professional development and promoted adoption of evidence based practices. For example, IBA promotes student retention by welcoming students in the annual conference and mentoring them into academic careers.

Faculty also need to be supported in efforts to create more inclusive and effective teaching environments. BioQUEST, IBA, and QUBES have made inclusive teaching, increasing diversity, and promoting equity core features of their activities through partnering with projects focused on these areas. For example, the Environmental Data Science Inclusion Network (EDSIN) project collaborates with QUBES and BioQUEST to support community discussions around inclusive practices in the emerging field of data science. This is an example of community driven efforts to shape the future of a new area in mathematical biology which supports a diverse professional community.

Faculty are preparing students for an unimagined future, equipping them for life-long learning in a rapidly changing world. The tools and technologies available for education and research are evolving at an ever increasing pace, and expectations for student experiences has evolved as well. Having students work in Excel has been replaced by having students conduct their analysis in R. Access to massive, real, messy data sets has replaced toy data sets, and with this comes a need for data science practices. Each of these reform organizations has grappled with this changing landscape, and they continue to be relevant by responding to the needs of the community and by looking ahead to identify the next education reform challenge. For example, the BioQUEST/QUBES 2019 summer workshop focused on data science, following the release of NASEM’s Data Science for Undergraduates report in 2018 National Academies of Sciences, Engineering, and Medicine (2018b). The goal of the workshop was to explore the impact of data science in biology and mathematics education. The definition of “student success” is ever changing, and effective educational reform efforts must keep pace to remain relevant.

*Systemic education reform efforts at the community level* The enormous success of the initiatives presented above has lessons for researchers and educators. It is difficult to quantify the impact of any professional development program on faculty practices, much less student outcomes (Condon et al. 2016). However, the impact of BioQUEST, MathBench, QUBES, and IBA is readily apparent in the community. Faculty surveys indicate that participants value the professional development experiences and resources offered by these groups. Anecdotally, the continued participation and commitment of faculty and institutions demonstrate both a need for these organizations and a high value on the resources they provide. Because these are grassroots organizations, they serve to empower the community to meet important goals in education. They are agile and responsive, but depend on volunteers to provide leadership and services. However, all of these projects require a baseline level of support for administrative efforts, cyber-platforms, and activities to effectively serve the community. BioQUEST, MathBench, QUBES, and IBA have explored a variety of business models which are influenced by each organization’s situation. NSF has provided a great deal of support over the years for these types of projects, and that funding has allowed these projects to reach out to the community and offer resources. However, competition for federal and foundation support is fierce and projects must diversify their approaches. MathBench was conceived and initially implemented at the University of Maryland and has since been funded to collaborate with faculty across the country and internationally. IBA began as a small collaboration between three institutions, and rapidly grew to a national collective using a membership model. The first instantiation of QUBES was an NSF funded Research Coordination Network in Undergraduate Biology Education incubator, which promptly merged with a wider effort emerging from the 2014 NSF Ideas Lab. QUBES now achieves the goal of providing educational materials and professional development to the community through contract services for educational projects and working with foundations such as Hewlett. BioQUEST began with a focus on curriculum development and expanded to professional development and supporting novel educational projects and over the decades has been supported by NSF, foundations such as Anneberg and HHMI, businesses such as Apple, and for fee professional development services. Funding education is an ongoing issue at all levels. Education reform projects might consider following the multi-institutional, interdisciplinary approach scientific research is currently taking. In recent years, there has been a very positive movement towards creating networks of institutions with similar educational aspirations (e.g. AAU STEM Education Initiative; NSF INCLUDES; NSF RCN UBE). These collaborative approaches provide an opportunity for coordination among individual institutions, allowing economies of scale, greater dissemination and adoption of effective practices, and broader support for reform. All four of the reform organizations highlighted here are built on collaborations. In an ideal world, mathematical biology education would play a major role in one of NSF’s funded research synthesis centers (Rodrigo et al. 2013).

## Conclusions

Rethinking how undergraduates are prepared for careers in research and the science and technology workforce requires careful consideration of the mathematical biology education system to identify key components and leverage points for change. Faculty, students, institutions, and resources are critical components of the system and efforts that support access to new resources, provide professional development with a holistic view of the student, and encourage appropriate career rewards are important contributions to successful change. A closer look at the four education reform organizations offered here provides an opportunity to examine how to leverage components of the education system including evolving student demographics, institutional expectations of faculty, access to resources through open education resources. These groups demonstrate that education reform remains relevant when it is rooted in and responsive to community-based efforts, and sustainable when organizations are flexible in exploring different organizational plans and funding options. As representatives of these organizations, the authors encourage readers to feel empowered to take steps to address their community’s needs, fill gaps, and explore new ideas through partnerships with BioQUEST, MathBench, QUBES, and IBA or by following their passion and launching a new initiative.
